# Recent Advances Regarding Polyphenol Oxidase in *Camellia sinensis*: Extraction, Purification, Characterization, and Application

**DOI:** 10.3390/foods13040545

**Published:** 2024-02-09

**Authors:** Chun Zou, Xin Zhang, Yongquan Xu, Junfeng Yin

**Affiliations:** 1 Key Laboratory of Biology, Tea Research Institute, Chinese Academy of Agricultural Sciences, Ministry of Agriculture and Rural Affairs, Hangzhou 310008, China; zouchun@tricaas.com (C.Z.); xinzhang@tricaas.com (X.Z.); 2 National Engineering Research Center for Tea Processing, Hangzhou 310008, China

**Keywords:** polyphenol oxidase, *Camellia sinensis*, extraction, purification, characterization, application

## Abstract

Polyphenol oxidase (PPO) is an important metalloenzyme in the tea plant (*Camellia sinensis*). However, there has recently been a lack of comprehensive reviews on *Camellia sinensis* PPO. In this study, the methods for extracting PPO from *Camellia sinensis*, including acetone extraction, buffer extraction, and surfactant extraction, are compared in detail. The main purification methods for *Camellia sinensis* PPO, such as ammonium sulfate precipitation, three-phase partitioning, dialysis, ultrafiltration, ion exchange chromatography, gel filtration chromatography, and affinity chromatography, are summarized. PPOs from different sources of tea plants are characterized and systematically compared in terms of optimal pH, optimal temperature, molecular weight, substrate specificity, and activators and inhibitors. In addition, the applications of PPO in tea processing and the in vitro synthesis of theaflavins are outlined. In this review, detailed research regarding the extraction, purification, properties, and application of *Camellia sinensis* PPO is summarized to provide a reference for further research on PPO.

## 1. Introduction

Polyphenol oxidase (PPO) belongs to the category of oxidoreductases and is widely present in plants [1], animals [2], and fungi [3]. According to the different numbers of phenolic hydroxyl groups in the catalytic substrate, PPO can be divided into three categories [4,5]: monophenol oxidase (tyrosinase, EC 1.14.18.1), bisphenol oxidase (catechol oxidase, EC 1.10.3.1), and laccase (EC 1.10.3.2). PPO in plants mainly occurs in the form of catechol oxidase, which can catalyze the generation of its corresponding quinones from polyphenols under aerobic conditions [6,7].

PPO plays an important role in tea processing [8,9], and it determines the degree of tea oxidation. According to the degree of oxidation, tea can be divided into six categories: green tea [10] (non-oxidized), white tea [11] and yellow tea [12] (lightly oxidized), oolong tea [13] (semi-oxidized), black tea [14] (fully oxidized), and dark tea [15] (post-fermented with micro-organisms). By inhibiting or promoting the enzymatic oxidation of PPO, various categories of teas with distinct flavors are produced. Under the catalysis of enzymes such as PPO, the catechins in tea are oxidized to form catechin polymers [16], including theasinensins, theaflavins, and thearubigins.

Tea plant PPO is encoded and expressed by nuclear genes [17], which have multi-gene family characteristics [18]. Zeng et al. [19] obtained five coding genes of PPO from the tea plant genome database with a total length of 597–1839 bp in the CDS region and encoding 198–612 amino acids. Through real-time quantitative PCR, it was found that these genes exhibit different expression patterns among different tea plant varieties. Many PPO isoenzymes with significant differences in their properties were isolated from fresh tea leaves. PPO in fresh tea leaves mainly exists as low activity precursor enzymes, which bind to organelle membranes such as chloroplasts in an insoluble form [20], which is known as membrane-bound PPO (mPPO). In addition, there are small amounts of mature enzymes, known as soluble PPOs (sPPOs), which have been removed from their transfer peptides and are free in a soluble form within the cystic body. There are differences in the extraction and purification of different types of PPO; this poses a challenge to tea plant PPO research.

Due to the enzymatic browning caused by PPO [21], researchers have focused on studying how to inhibit PPO activity in other plants or fungi. However, the catalytic activity of PPO needs to be utilized in the processing of tea (except for unfermented tea) and the preparation of theaflavins. Therefore, it is necessary to elaborate in detail on the tea plant PPO. In this study, detailed research on the extraction, purification, properties, and application of *Camellia sinensis* PPO is summarized to provide a reference for further research on PPO.

## 2. Extraction of PPO

The methods for extracting PPO from fresh tea leaves include acetone extraction, buffer extraction, and surfactant extraction. The extraction solvents, as well as the advantages and disadvantages of the above three methods, are listed in Table 1.

### 2.1. Acetone Extraction

The advantages of the acetone extraction method [29] are that it has high enzyme activity and can be directly applied; moreover, the enzyme is stable and easy to store. However, its disadvantage is that the enzyme extraction rate is too low, possibly due to acetone causing irreversible protein denaturation. To reduce this enzyme denaturation, acetone needs to be pre-cooled before use. Frozen tea leaves were homogenized in cold acetone (−25 °C), and the slurry was subjected to repeated filtration and cold acetone extraction to obtain a white crude enzyme powder [23]. The acetone extraction method has been applied to analyze the changes in PPO activity of different varieties of tea leaves during black tea processing [22]. In addition, this method has also been widely used for PPO extraction in other plants, including apple [29], *Physalis peruviana* L. [30], and *Cistanche deserticola* [31].

### 2.2. Buffer Extraction

The buffer extraction method is used to obtain PPO by mixing tea leaves with buffer and homogenizing them, then filtering them out. This method has the advantages of simple operation and low impurities in the enzyme solution. However, the PPO activity extracted by this method is relatively low, and the extraction solution needs to be further concentrated and purified before it can be applied. As shown in Table 1, the specific activity of PPO extracted by buffer is 192 U/mg, which is significantly lower than that extracted by acetone or surfactant. The types of buffers used in this method include phosphate buffer (pH 6.8) [24] and citric acid phosphate buffer (pH 5.6) [25]. In order to reduce the content of tea polyphenols in the extraction solution, polyvinyl pyrrolidone (PVP) and cross-linked polyvinylpyrrolidone (PVPP) are added to the buffer to adsorb polyphenols. It was found that the activity of PPO obtained by adding PVP was higher than that of PVPP, possibly due to the fact that PVP, with its good water solubility, can adsorb more tea polyphenols [25]. The buffer extraction method is widely used for the extraction of soluble PPO from different plants [32,33], but it cannot extract the membrane-bound PPO, resulting in a lower extraction rate.

### 2.3. Surfactant Extraction

Non-ionic surfactants mainly rely on hydrophobic interactions to dissolve membrane proteins, which are usually used for the extraction of membrane-bound PPO in plants [34,35,36]. The non-ionic surfactant used for extracting membrane-bound PPO from tea leaves is usually Triton X-100. The surfactant is dissolved in a buffer at a certain concentration (usually 50 mM) of salt ions, which contributes to stabilization of the enzyme protein. The fresh leaves of three tea tree varieties (Ningzhou population, Ningzhou 2, and Dayelong) were homogenized with phosphate buffer (pH 6.8) and centrifuged to obtain the supernatant containing soluble PPO. Then, the precipitate was extracted with 0.25% Triton X-100 to obtain membrane-bound PPO [26]. The surfactant extraction method can achieve a higher PPO extraction rate, but the addition of surfactants may interfere with the determination of enzyme properties.

## 3. Purification of PPO

**Figure 1:**
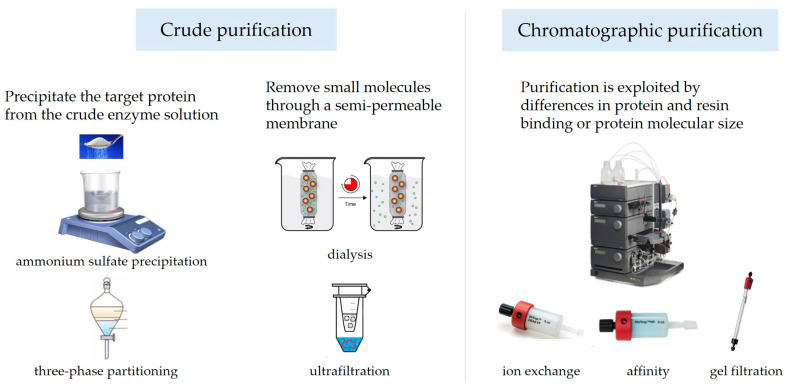
The main purification steps for *Camellia sinensis* PPO.

The crude enzyme solution extracted from tea leaves contains not only nucleic acids, polyphenols, etc., but also other proteins in addition to PPO. As shown in Figure 1, the purification of PPO is generally divided into two major steps: crude purification and fine purification. The crude separation of PPO mainly includes ammonium sulfate precipitation [37], three-phase partitioning [38], dialysis [39], and ultrafiltration [40]. The fine purification of PPO is generally carried out via chromatography, including ion exchange chromatography [41], gel filtration chromatography [42], affinity chromatography [43], etc.

### 3.1. Crude Purification

#### 3.1.1. Ammonium Sulfate Precipitation

The principle behind the ammonium sulfate precipitation method [44] is that high concentrations of salt ions can compete with proteins for water molecules, thereby destroying the hydration film on the surface of proteins, reducing their solubility, and allowing them to precipitate out of the solution. This method can remove a large amount of non-protein impurities and also concentrate the target protein. After a crude enzyme solution extracted from *Camellia sinensis* cv. Longjing 43 was precipitated with 80% ammonium sulfate, the specific activity of PPO was found to increase by 3.73-fold [45].

Graded ammonium sulfate precipitation is commonly used to remove some impurity proteins, and its principle is based on the difference in protein solubility in different ammonium sulfate concentrations. PPO crude enzyme was added with 10%, 20%, 30%, 70%, 80%, and 90% of ammonium sulfate in sequence and then left to stand at 4 °C to precipitate the proteins. While testing the enzymatic activities of the precipitated proteins mentioned above, it was found that the enzymatic activity of PPO proteins precipitated with 10–30% ammonium sulfate was very low (<3%), while that of proteins precipitated with 30–90% ammonium sulfate reached 65.26% of the total enzyme activity [46]. Therefore, it is important to select an appropriate concentration of ammonium sulfate to precipitate proteins during the crude separation of PPO.

#### 3.1.2. Three-Phase Partitioning

Three-phase partitioning (TPP) is a method of crude purification of target proteins, which involves adding a certain proportion of salt and organic solvents to the crude extraction solution to create clear layering of the mixed solution [47]. This method promotes the aggregation of some of the proteins in the precipitation layer between the organic and aqueous phases, the dissolution of low-molecular-weight pigments, membrane lipids, etc., in the organic layer, and the dissolution of sugars and some proteins in the water layer. The sPPO and mPPO from tea leaves were purified via TPP, resulting in 2.80-fold and 2.32-fold increases in specific enzyme activity, respectively [26]. The activity yields of TPP to sPPO and mPPO were 73.8% and 79.8%, respectively. With its advantages of simple operation, wide applicability, and high activity yield, TPP has been widely used in the extraction of PPO from various plants, including *Rosmarinus officinalis* L. [47], *Lepiota procera* [48], and *Trachystemon orientalis* L. [38]. The PPO from *Trachystemon orientalis* L. was purified 3.59-fold with a 68.75% total recovery of activity using the TPP procedure twice in a row [38].

#### 3.1.3. Dialysis

As a result of methods such as ammonium sulfate precipitation or three-phase partitioning, large amounts of salt ions are introduced into the enzyme solutions, requiring dialysis to remove them. Dialysis is a method of separating proteins and small molecules utilizing small molecules to penetrate through a semi-permeable membrane into a low salt buffer while large molecules, such as proteins, remain trapped within the semi-permeable membrane [49]. Usually, the sample is placed in a dialysis bag made of a semi-permeable membrane, and the dialysis bag is immersed in a low salt buffer solution. Salt and small-molecule substances are used to continuously diffuse and dialyze outside the bag, achieving purification [50]. To achieve good purification results, the low salt buffer needs to be replaced multiple times. Following ammonium sulfate precipitation, the PPO from fresh tea leaves was dialyzed in a cut-off with 8–12 KDa, and the purification factor was found to increase by 2.42 times [51].

#### 3.1.4. Ultrafiltration

Ultrafiltration can achieve high concentration multiples, making it easy to concentrate and recover the target product from diluted and complex mixed samples [52]. It is necessary to select ultrafiltration tubes, which retain molecular weight based on the molecular weight of the target protein [53]. Rapidly reducing salt ions in samples can also be achieved through ultrafiltration. Following TPP treatment, sPPO and mPPO in fresh tea leaves were centrifuged through an ultrafiltration tube (molecular weight cut-off of 15 kDa) using centrifugal force of 4500× *g* at 4 °C, and their purification times were found to increase by 9.58-fold and 9.05-fold, respectively [27].

### 3.2. Chromatographic Purification

Chromatographic chromatography is generally used for fine purification of enzyme proteins after crude purification [54]. As shown in Table 2, the chromatographic methods used for PPO purification from tea sources mainly include ion chromatography, gel filtration chromatography, and affinity chromatography. In order to achieve good purification results, it is very important to choose the appropriate resin and elution buffer [55].

#### 3.2.1. Ion Exchange Chromatography

The principle behind ion exchange chromatography is that the charge carried by the separated substance can combine with the opposite charge carried by the ion exchange agent [58]. The binding effect between the charged molecule and the stationary phase is reversible. When changing the pH or eluting with a buffer solution, which gradually increases the ion strength, the substance bound by the ion exchange agent can exchange with the ions in the eluent and be eluted into the solution [59]. Due to differences in the charges of different proteins, their binding abilities to ion exchangers also vary, resulting in different orders of elution into the solution [60]. The resins, which have been used for ion exchange chromatography in the purification of PPO, are DEAE-cellulose [61] and UNOsphere™ Q (BioRad, Hercules, CA, USA) [62]. The pH of the buffer solution for both DEAE and Q ion exchange chromatography needs to be at least one unit higher than the pI of the target protein to be bound, where the pH of the buffer solution for Q ion exchange chromatography is higher than that of DEAE. Based on the amino acid sequence of PPO published in the NCBI database, the pI of most tea tree PPOs is predicted to be about pH 6.4. PPO was separated by linearly increasing the buffer solution from a low salt ion concentration to a high salt ion concentration. The purification fold results of PPO purified using DEAE and Q ion exchange chromatography were found to be 3.32 [23] and 11.8 [56], respectively, which indicates that it is difficult to obtain high-purity PPO solely through ion exchange chromatography.

#### 3.2.2. Gel Filtration Chromatography

Gel filtration chromatography [63], also known as steric exclusion chromatography and molecular sieves, is a method of separating the proteins based on their differences in molecular weight or shape. In order to obtain a good purification effect, it is necessary to select a chromatographic matrix with a pore size, which is suitable for the molecular weight of the target protein [64]. Sephadex G-75 was used as a chromatographic substrate for the purification of PPO from tea leaves, and a purification fold of 48.94 was achieved [51]. Therefore, the protein can be highly purified by gel filtration chromatography. Gel filtration chromatography is widely used for the purification of PPO from other sources, including *Coriandrum sativum* [65], *Musa acuminata* [66], and sweet potato [42]. Two sPPO and one mPPO from sweet potato peel [42] were purified by gel filtration chromatography with the purification fold of 69.03, 31.59, and 124.01, respectively.

#### 3.2.3. Affinity Chromatography

Affinity chromatography is a protein purification method, which is designed based on the specific and reversible binding between proteins and matrices [43]. Nickel column affinity chromatography is a widely used method for purifying recombinant proteins [67]. Due to the competitive binding of Ni^2+^ ions in nickel columns to imidazole or proteins with His-Tag, increasing the concentration of imidazole in the elution buffer can elute the target protein to achieve protein purification. Two PPO isoenzymes with His-Tag expressed by *Escherichia coli* were purified via binding to an Ni IDA affinity chromatography column and eluting with different concentrations of imidazole (25–500 mM) [57]. PPO from tea leaf was purified 19.77-fold in one step using Sepharose 4B-L-tyrosine-p-aminobenzoic acid affinity chromatography [28]. Compared to other chromatography methods, affinity chromatography has the advantages of simplicity and speed. Sepharose 4B-L-tyrosine-p-aminobenzoic acid and Sepharose-6B-L-tyrosine-p-aminobenzoic acid were applied to the affinity chromatography of PPO from *Persea americana* [43], which obtained the purification fold of 147.73 and 154.00, respectively.

## 4. Characterizations of PPO

The characterizations of PPO from different sources of tea plants were systematically compared in terms of optimal pH, optimal temperature, molecular weight, substrate specificity, and activators and inhibitors. As shown in Table 3, PPO characterizations vary not only among different *Camellia sinensis* varieties but also among different isoenzymes derived from the same tea leaves.

### 4.1. Optimal pH of PPO

In Table 3, the optimal pH values of PPO from different tea leaves are reported, varying between 5.0 and 6.2. The reason for the difference in the optimal pH of PPO from different tea plants may be its different structures, especially PPOs with large molecular weight differences. The optimum pH of PPO from tea leaves in Turkey was found to be 6.0 [23,68]. Different PPO isoenzymes isolated from tea leaves show differences at the optimal pH. Two PPO isozymes from *Camellia sinensis* var. Zhenghedabai were purified [51], with one PPO isozyme having an optimal pH of 6.0 and the other PPO isozyme having an optimal pH of 5.5. There are significant differences in the optimal pH of PPO from different plant sources [70]. The PPO from tea leaves with similar optimal pH levels includes *Vaccinium corymbosum* L. [71], Ataulfo mango [72], and *Solanum lycocarpum* [73].

### 4.2. Optimal Temperature of PPO

The optimal temperature of PPO from different tea leaves is mostly in the range of 30–38 °C. The catalytic activity of PPO is highest at the optimal temperature, and it decreases above or below the optimal temperature [74]. The optimal temperature for PPO varies among different tea varieties, as well as among the same variety of isoenzymes. The optimal temperature for one type of PPO isoenzyme from Huangjinya tea was determined to be 35 °C, while that for another type of PPO isoenzyme was 30 °C [57]. The optimal temperature for PPO in tea leaves is similar to that for some other plants, such as *Dioscorea alata* [75], *Terfezia arenaria* [76], and *Salacca zalacca* [77].

### 4.3. Molecular Weight of PPO

The molecular weight of PPO in tea leaves has been reported to range from 15 to 97 kDa [56,78]. Currently, there are 36 protein sequences of PPO from *Camellia sinensis*, which can be retrieved from the NCBI database, most of which have 599 amino acids. Based on the number of amino acids, it has been inferred that the molecular weight of most PPOs from *Camellia sinensis* is approximately 66 kDa. The PPO from *Camellia sinensis* var. Lapsang souchong was isolated from a black tea infusion, and its molecular weight was determined to be 66 kDa [56]. Five *ppo* genes from five cultivars of *Camellia sinensis* were expressed in *E. coli* BL21, and all of the five recombinant PPOs obtained exhibited molecular weights of 66 kDa [79].

Due to the presence of many PPO isoenzymes in tea plants, there are differences in the molecular weight of PPO reported in different studies. Two PPO isozymes were isolated from tea leaves [51], and their molecular weights were found to be 42 and 85 kDa, respectively. There are also differences in the molecular weight of PPOs derived from different plants. PPO in *Pueraria lobata* was purified [80], and its molecular weight was determined to be 21 kDa via SDS-PAGE. The molecular weight of PPO from Huaniu Apples [81] was determined to be 140 kDa using native-PAGE and SDS-PAGE, but on the basis of urea-SDS-PAGE, it was found to be 61 kDa, which indicates that it may be a dimer. The high abundance of the PPO homodimer suggests that it may be involved in proanthocyanidins polymerization, which leads to the formation of the dark-red skin of apples. However, it has not been reported whether PPO from *Camellia sinensis* is a polymer.

### 4.4. Substrate Specificity of PPO

The substrates used for PPO include catechol, 4-methyl catechol, catechins, pyrogallol, and gallic acid [6,7]. Among them, catechol is the most widely used substrate. Eight substances were used to test the substrate specificity of purified PPO [69]. Among them, three substances—p-quinol, p-cresol, and tyrosine—cannot be catalyzed by PPO; meanwhile, the other five substances—catechin, epicatechin, catechol, pyrogallol, and gallic acid—can be used as substrates for PPO. The Km value for catechin is the lowest, indicating that it has the highest affinity with PPO. Altunkaya [68] found that PPO not only had the highest affinity for catechin, but it also had the highest catalytic efficiency toward it, taking into account the highest Vmax/Km ratio. There is a significant difference in substrate specificity between PPO from tea leaves and other plants. PPO from *Irvingia gabonensis* [82] was found to show preference toward catechol, with a relative activity of 100%; on the other hand, it had lower catalytic activity toward catechin, with a relative activity of 77.1%.

### 4.5. Activators and Inhibitors of PPO

Due to the presence of two Cu^2+^ binding regions in the active center of PPO, Cu^2+^ is considered an activator of PPO [83]. Testing of the effect of different Cu^2+^ concentrations on the activity of *Camellia sinensis* PPO [78] showed that it had the highest catalytic activity when the Cu^2+^ concentration was 10^−7^ M. Although SDS, urea, and surfactants have been reported to activate some plant PPOs [5,84], they are considered to have no activating effect on *Camellia sinensis* PPO [69]. The purified PPO and crude enzyme extracts from tea leaves were treated with SDS (0.1–5 mM) and urea (0.5–2 M), but no activation effect was detected.

The inhibitors of *Camellia sinensis* PPO include sodium metabisulfite, sodium sulfite, ascorbic acid, EDTA, cysteine, citric acid, and oxalic acid [23,28]. However, there are differences in the inhibitory effects of inhibitors on PPO from different tea leaves. The inhibitory effect of cysteine on PPO from *Camellia sinensis* var. Lapsang souchong [56] was found to be stronger than that of ascorbic acid, while ascorbic acid was found to be the most effective inhibitor of PPO from Turkish tea leaves [68], followed by cysteine. Both cysteine and ascorbic acid have been determined as competitive inhibitors of PPO.

## 5. Application of PPO

Controlling the PPO activity in tea processing greatly affects its quality, especially in tea with high fermentation levels, such as black tea and dark tea. In addition, PPO is widely used in the in vitro synthesis of theaflavins.

### 5.1. The Role of PPO in Tea Processing

As shown in Table 4, there is a substantial difference between PPO in black tea processing and dark tea processing. In black tea processing, PPO comes from endogenous enzymes in fresh tea leaves. However, endogenous enzymes are inactivated in the first step in the processing of dark tea, while PPO is produced by micro-organisms in subsequent processes. The catalytic effect of PPO is present in the fermentation of black tea and in the pile fermentation of dark tea. In addition, the products of PPO oxidation are mainly theaflavins and thearubigins in black tea, but theabrownines in dark tea.

#### 5.1.1. Black Tea

Black tea is fully fermented tea, and during its processing, it produces theaflavin pigments through enzymatic oxidation of catechins, forming its unique color and aroma [85]. PPO is a key enzyme in the enzymatic oxidation of black tea, and its enzyme activity dynamically changes during processing. In black tea processing, PPO activity increases during withering and rolling processes, while it decreases during fermentation and drying processes. PPO activity was found to increase with the prolongation of withering time during withering [86]; at the end of withering, PPO activity reached a level, which was 2.9 times that of fresh leaf PPO activity. Rolling can cause damage to the tea leaves through external forces, resulting in polyphenolic compounds, endogenous PPO, and other components leaking into the leaf epidermis and coming into full contact with oxygen and other substances. PPO activity was found to reach its highest level during the rolling process [87]. Fermentation is a key process in forming the quality characteristics of black tea, which is essentially a chemical change process, which occurs through enzymatic or non-enzymatic oxidation reactions with polyphenolic compounds. The fermentation process of black tea is influenced by various factors, such as oxygen [88], temperature [89], humidity [85], and fermentation time [90]. Oxygen [88] was found to be the key factor limiting the oxidation rate of polyphenols in regular black tea fermentation. A low fermentation temperature [89] was beneficial to promoting the accumulation of theaflavins and thearubigins. Under different temperature conditions, it was found that PPO activity in all samples decreased significantly with fermentation [91]. Drying rapidly deactivates various enzymes in tea due to the high temperature [92].

Enhancing PPO activity in black tea processing is an effective way of improving the quality of black tea. Comparing the processing of fresh tea leaves from different varieties, seasons, and regions for Congou Black Tea [93], it was found that the black tea obtained from processing fresh tea leaves with high PPO activity had a higher content of theaflavins. It was found that oxygen was consumed in large quantities during the processing of black tea [94]. Compared with traditional fermentation methods, a new dynamic fermentation method has been developed, which effectively improves PPO activity by increasing the oxygen content during the fermentation process [95], thereby promoting the formation of theaflavins and thearubigins and improving the quality of black tea. Moreover, oxygen-enriched fermentation [96] was found to improve the taste of black tea and promote the oxidation of catechins, flavonoid glycosides, and some phenolic acids. In addition, adding exogenous PPO [97,98] to black tea processing improves the color and aroma of its tea soup and also increases the content of theaflavins.

**Table 4:** Comparison of PPO in black and dark tea processing.

Tea Category	PPO Source	Enzyme-Catalyzed Process	Products	References
Black tea	Endogenous enzymes in fresh tea leaves	Fermentation	Theaflavins and thearubigins	[14,85]
Dark tea	Microbial secretion	Pile fermentation	Theabrownines	[99,100,101]

#### 5.1.2. Dark Tea

**Figure 2:**
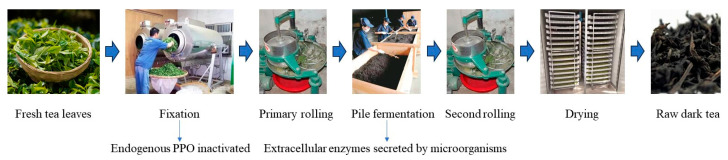
Primary processing of dark tea.

Dark tea is a type of post-fermented tea [99]. As shown in Figure 2, the processing of black tea mainly includes fixation, primary rolling, pile fermentation, second rolling, and drying. During the fixation process, the endogenous enzymes in tea leaves are inactivated. The key process in dark tea processing is pile fermentation. In this process, micro-organisms proliferate in large numbers and secrete extracellular enzymes, such as PPO, protease, and cellulase, which form the unique flavor and quality of dark tea [100]. During the processing of Fuzhuan brick tea [101], there is a trend in PPO activity to initially increase and then decrease, which is significantly correlated with the growth curve of *Eurotium cristatum*. It was found that adding exogenous PPO to the pile fermentation of Pu-erh tea [102] can accelerate its fermentation, shorten the fermentation cycle, and improve its quality.

### 5.2. Synthesis of Theaflavins by PPO

**Figure 3:**
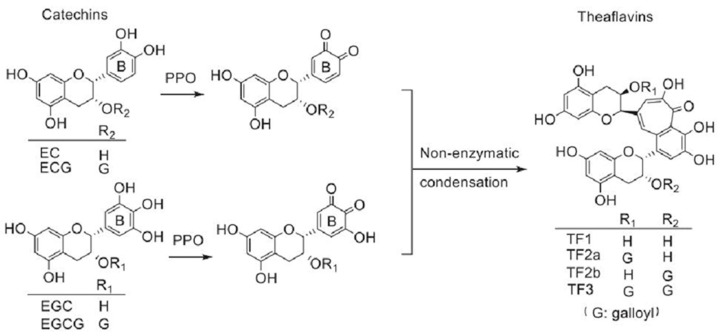
The formation of four theaflavins (TF1, TF2a, TF2b, and TF3) [104].

Theaflavins are a type of plant pigment formed by the oxidation and condensation of catechins [103], which have various health benefits [104], such as anti-obesity, anti-inflammatory, and anti-cancer properties. Theaflavins are a key quality component of black tea and are mainly present in four forms (Figure 3): theaflavin (TF1), theaflavin-3-*O*-gallate (TF2a), theaflavin-3′-*O*-gallate (TF2b), and TF-3,3′-di-*O*-gallate (TF3) [104]. Catechins are first oxidized by PPO enzyme to form quinones, which are further oxidized to form theaflavins through non-enzymatic oxidation. However, TFs only account for about 1% of the dry weight of black tea, and the direct extraction cost is too high. Therefore, enzymatic oxidation through PPO in vitro is a more efficient and economical method for producing theaflavins [105,106]. Two types of PPO isoenzymes were isolated from fresh tea leaves and used for in vitro synthesis of theaflavins [51]. Among them, one PPO isoenzyme only resulted in the synthesis of simple TF, while the other isoenzyme could synthesize four types of TFs. Recombinant expression through micro-organisms is an important method for obtaining tea plant PPO [107]. Four tea plant PPO isoenzymes were prepared via recombinant expression in *E. coli* [108]. Although most of the recombinant enzymes exist as inclusion bodies, they can still efficiently catalyze the synthesis of TF3.

## 6. Conclusions and Perspectives

In this study, the isolation, purification, properties, and applications of tea plant PPO were systematically reviewed. The methods for extracting PPO from fresh tea leaves include acetone extraction, buffer extraction, and surfactant extraction, and their advantages and disadvantages were compared. The crude separation and fine purification methods of PPO and their purification effects were introduced in detail. The characterizations of PPO from different sources of tea plants were systematically compared in terms of optimal pH, optimal temperature, molecular weight, substrate specificity, and activators and inhibitors. The applications of PPO in tea processing and theaflavin synthesis were summarized.

Although significant achievements have been made in the research and application of tea plant PPO, there are still several aspects worth further investigation in the following areas: (1) the crystal structure of tea plant PPO protein needs to be detected. Currently, researchers have successfully purified PPO from multiple varieties of tea trees. If the protein structure of PPO can be further detected, this will help further explore its catalytic mechanism. (2) Further improvements are needed for the preparation of PPO through microbial recombinant expression. Currently, the main factor restricting widespread application of PPO is the difficulty in obtaining low-cost and highly active enzymes. The efficient recombinant expression of PPO through genetic engineering will greatly expand its application scope.

## Figures and Tables

**Table 1:** Comparison of extraction methods for *Camellia sinensis* PPO.

Method	Extract Solvent	Specific Enzyme Activity (U/mg)	Advantage	Disadvantage	References
Acetone extraction	Acetone	24,789	High enzyme activity, stable and easy to store	Low extraction rate of enzyme	[22,23]
Buffer extraction	Phosphate/citrate buffer	192	Easy operation, less impurity	Low enzyme activity and extraction rate	[24,25]
Surfactant extraction	Triton X-100	20,544	High extraction rate of enzyme	Surfactant needs to be removed	[26,27,28]

**Table 2:** Comparison of chromatographic chromatography for *Camellia sinensis* PPO.

Type of Chromatography	Chromatographic Matrix	Elution Buffer	Purification Fold	References
Anion exchange	DEAE-cellulose	A linear gradient of phosphate buffer (pH 6.8) concentration from 10 to 200 mM	3.32	[23]
Anion exchange	UNOsphere™ Q	A linear concentration gradient (0–1.0 M) of NaCl in 20 mM Tris-HCl (pH 9.0)	11.8	[56]
Gel filtration	Sephadex G-75	0.02 M Tris–HCl buffer (pH 7.5) containing 100 mL/L glycerol and 0.1 M NaCl	48.94	[51]
Affinity	Ni-NTA	Imidazole solution of 25–500 mM	Unknown	[57]
Affinity	Sepharose 4B-L-tyrosine-p-aminobenzoic acid	0.1 M Tris–HCl buffer (pH 8.5) containing 1 M NaCl	19.77	[28]

**Table 3:** Comparison of *Camellia sinensis* PPO characterizations.

Source	pH	Temperature (°C)	Molecular Weight (kDa)	References
Two PPO isozymes from *Camellia sinensis* var. Zhenghedabai	5.5 and 6.0	33 and 38	85 and 42	[51]
PPO from *Camellia sinensis* var. Lapsang souchong	6.2	35	66	[56]
PPO from Turkish tea leaves	6.0	30	72	[23]
PPO from Turkish tea leaves	6.0	30	Unknown	[68]
PPO from Indian tea leaves	5.0	Unknown	72	[69]
Two recombinant PPO isozymes from Huangjinya tea	6.0 and 5.5	35 and 30	61.15 and 61.21	[57]

## Data Availability

Data is contained within the article.
